# A novel screening protocol for the isolation of hydrogen producing *Chlamydomonas reinhardtii *strains

**DOI:** 10.1186/1471-2229-8-107

**Published:** 2008-10-17

**Authors:** Thilo Rühle, Anja Hemschemeier, Anastasios Melis, Thomas Happe

**Affiliations:** 1 Fakultät für Biologie und Biotechnologie, Lehrstuhl für Biochemie der Pflanzen, AG Photobiotechnologie, Ruhr-Universität Bochum, 44780 Bochum, Germany; 2 Department of Plant and Microbial Biology, University of California, Berkeley, California, 94720-3102, USA

## Abstract

**Background:**

Sealed *Chlamydomonas reinhardtii *cultures evolve significant amounts of hydrogen gas under conditions of sulfur depletion. However, the eukaryotic green alga goes through drastic metabolic changes during this nutritional stress resulting in cell growth inhibition and eventually cell death. This study aimed at isolating *C. reinhardtii *transformants which produce hydrogen under normal growth conditions to allow a continuous hydrogen metabolism without the stressful impact of nutrient deprivation.

**Results:**

To achieve a steady photobiological hydrogen production, a screening protocol was designed to identify *C. reinhardtii *DNA insertional mutagenesis transformants with an attenuated photosynthesis to respiration capacity ratio (P/R ratio). The screening protocol entails a new and fast method for mutant strain selection altered in their oxygen production/consumption balance. Out of 9000 transformants, four strains with P/R ratios varying from virtually zero to three were isolated. Strain *apr*1 was found to have a slightly higher respiration rate and a significantly lower photosynthesis rate than the wild type. Sealed cultures of *apr*1 became anaerobic in normal growth medium (TAP) under moderate light conditions and induced [FeFe]-hydrogenase activity, yet without significant hydrogen gas evolution. However, Calvin-Benson cycle inactivation of anaerobically adapted *apr*1 cells in the light led to a 2-3-fold higher *in vivo *hydrogen production than previously reported for the sulfur-deprived *C. reinhardtii *wild type.

**Conclusion:**

Attenuated P/R capacity ratio in microalgal mutants constitutes a platform for achieving steady state photobiological hydrogen production. Using this platform, algal hydrogen metabolism can be analyzed without applying nutritional stress. Furthermore, these strains promise to be useful for biotechnological hydrogen generation, since high *in vivo *hydrogen production rates are achievable under normal growth conditions, when the photosynthesis to respiration capacity ratio is lowered in parallel to down regulated assimilative pathways.

## Background

Oxygen is a key regulator of the switch between the two different worlds, photosynthetic growth and anaerobic life, of *C. reinhardtii*. The green alga has an outstanding ability to adapt its metabolism to oxygen availability [1-3]. Under normal growth conditions, *C. reinhardtii *wild type cultures exhibit a four to seven fold higher photosynthesis than respiration rate. Anabolic reactions like carbon dioxide fixation and protein biosynthesis predominate during net oxygen production. Photolytically evolved oxygen is also used for respiration providing the metabolism with a further source of ATP besides photophosphorylation. Hydrogen production does not take place, since the hydrogenase genes are not expressed under aerobic conditions [4] and the oxygen-sensitive [FeFe]-hydrogenases would be inhibited by photosynthetically evolved oxygen [5,6].

Under laboratory conditions, anoxia can be established by flushing cultures with argon or by incubating sealed algal cells in the dark. As soon as any dissolved oxygen is removed, fermentative reactions are activated in order to maintain the NAD/NADH balance and ATP supply [1,7]. Under such conditions, the [FeFe]-hydrogenase gene expression is triggered [8]. When anaerobically adapted algal cultures are shifted to sudden illumination, a short term hydrogen production can be observed [9]. The [FeFe]-hydrogenase HydA1 transiently accepts electrons from photosynthetically reduced ferredoxin PetF. This phenomenon can be measured as a short hydrogen production flash. Soon, oxygen production by PSII inhibits the hydrogenase, and photosynthetically generated electrons are consumed in the re-activated Calvin-Benson cycle [10].

The antagonism between oxygenic photosynthesis and oxygen-sensitive hydrogen production can be circumvented by exposing algal cells to nutritional stress. Long term hydrogen production in the light is established when *C. reinhardtii *cultures are transferred to sulfur-depleted medium [11]. The absence of sulfur leads to decline of photosynthetic activity with photosystem II (PSII) being the primary target. The oxygen production rate drops below the respiration rate after one or two days of sulfur deprivation and the algal culture becomes microaerobic/anaerobic. Then, [FeFe]-hydrogenase genes are expressed and residual H_2_O photolytic activity acts as one important electron source for hydrogen production [12]. Under such conditions of nutritional and anaerobic stress, the release of H_2 _permits low levels of photophosphorylation and thus the continuous generation of ATP [13,14]. This energy source (ATP) ensures survival of the cells for a prolonged period of time under the above-mentioned adverse conditions. Beside the residual PSII activity, endogenous starch metabolism plays a significant role as electron source for hydrogen formation [15-17]. In the aerobic phase of sulfur deprivation, cells accumulate up to 8-fold more starch than under normal growth conditions [11]. When the photosynthesis/respiration ratio (P/R ratio) drops below a ratio of one and anaerobic conditions are established, fermentative pathways are induced including starch degradation. The reduction of the plastoquinone pool is driven by a NAD(P)H plastoquinone-oxidoreductase (NDH2) which oxidizes reducing equivalents originating from starch and protein degradation [18]. As a consequence, the proton gradient across the thylakoid membrane is maintained and photophosphorylation can still occur, although PSII activity is low.

This work describes an alternative approach for the induction of hydrogen metabolism with mutants attenuated in their photosynthesis/respiration capacity ratio [19]. By attenuating the P/R ratio to a level lower than one, the culture becomes anaerobic in the light. Attenuated P/R ratio mutants (*apr *mutants) are expected to mimic the physiological status of sulfur-deprived cells. However, these mutants allow the analysis of endogenous substrate pathways and the expression of hydrogen metabolism associated genes under steady state growth conditions without the background of nutritional stress. Furthermore, such strains could constitute a basis for continuous hydrogen generation in biotechnological approaches.

This study presents the physiological analysis of attenuated P/R ratio strains (*apr*), which were identified with a novel screening protocol comprising a high-throughput application of the Winkler test. It is demonstrated that one of the isolated mutant strains, *apr*1, induces a hydrogen metabolism under normal nutrient growth conditions in the light. After Calvin-Benson cycle inactivation, hydrogen production rates of the *apr*1 strain were twice as high as those reported for the sulfur-depleted wild type.

## Results

### Mutant strain library construction

The mutant library was generated by DNA insertional mutagenesis in two different approaches. In the first approach, a cell wall-less, arginine auxotrophic strain (CC-425) was used as recipient strain and was transformed with the *Hind*III-linearized pJD67 plasmid carrying the intact *arg*7 gene [20]. In the second approach, the wild type strain CC-124 was employed as background strain and transformed with a paromomycine resistance cassette derived from plasmid pSL18 [21]. About 3000 mutants from the CC-425 and about 6000 mutants from the CC-124 background strain were generated and subjected to the below-mentioned screening procedure.

### Winkler test screen and its validation

A common screening protocol to isolate *C. reinhardtii *mutants with impaired photosynthesis entails the detection of acetate requirement, thus the isolation of mutants which are not able to grow photoautotrophically [22]. This type of screening, which selects for mutants with severe defects in the photosynthetic metabolism, was not applicable for our approach, since we aimed to analyze steady state hydrogen production, and a significant hydrogen generation by *C. reinhardtii *depends on photosynthetic activity [1,13,23]. Thus, we did not screen for mutant strains with drastic defects of photosynthesis, but our screening procedure had to differentiate between oxygen-producing transformants and transformants which become anaerobic in the light. Therefore, the Winkler test, which determines water-dissolved oxygen concentrations in four chemical reactions (Table 1), was applied [24]. In principle, dissolved oxygen is fixed in a redox reaction with manganese chloride. After an acidification step, added iodide ions are oxidized to iodine in stoichiometric amounts to dissolved oxygen. The iodine concentration can be measured by titrating with sodium thiosulfate. The molar amount of added sodium thiosulfate is therefore equivalent to dissolved oxygen concentrations. Starch solution is supplemented as a visual indicator to simplify the identification of the titration point.

**Table 1:** Overview of the Winkler test reactions which allow the quantification of dissolved oxygen.

	***Reaction***	***Comment***
1) oxygen fixation	Mn^2+ ^+ 2OH^- ^→ Mn(OH)_2_ 4Mn(OH)_2 _+ O_2 _+ 2H_2_O → 4Mn(OH)_3 _(s)	Dissolved oxygen is fixed with manganese chloride
2) Manganese dissolving	2Mn(OH)_3 _+ 2I^- ^+ 6 H^+ ^→ 2Mn^2+ ^+ I_2 _+ 6H_2_O	Acidifying the solution by adding H_3_PO_4 _dissolves the brown precipitate. Mn(III) cations are liberated which oxidize added iodide to iodine
3) Starch addition		Elementary iodine intercalates within starch helices turning the solution into a dark-blue color
4) Titration	I_2 _+ 2S_2_O_3_^2- ^→ 2I^- ^+ S_4_O_6_^2-^	The oxygen concentration in each well can be determined by titrating with sodium thiosulfate

The test comprises four steps in which MnCl_2_, KI/NaOH, H_3_PO_4 _and starch solutions are added successively into the test wells. Cultures which stay anaerobic can be determined colorimetrically.

It was important that all cells were in the linear or exponential growth phase, because oxygen production rates for the wild type decline in the stationary growth phase. Measurements of non-growing cells would result in a high number of false positives. For that reason, each transformant was supplied with fresh TAP medium six hours before the test was performed to ensure oxygen production of transformants with normal P/R ratios. The first two reaction steps were carried out under anaerobic conditions to prevent diffusion of atmospheric oxygen into the samples. For sufficient oxygen production, the plates were incubated in an anaerobic tent or in a nitrogen gas-flushed glove box about half an hour under 60 to 80 μE × m^-2 ^× s^-1 ^incident actinic light. The subsequent steps of the Winkler test could be performed under aerobic conditions, because the acidification step prevents oxygen fixation by manganese chloride, the latter occurring only under basic conditions.

**Figure 1:**
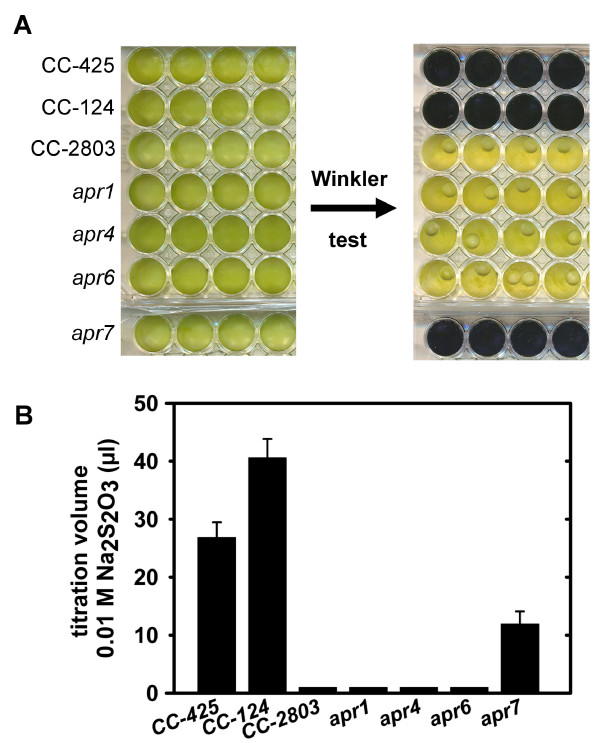
**Winkler test with mutant strains producing low amounts of oxygen**. Wild types CC-425 and CC-124 served as positive controls, the Rubisco-deficient strain CC-2803 as a negative control. A) Detail of a 48-well plate with 1 ml algal cultures before (left) and after MnCl_2_, KI/NaOH, H_3_PO_4 _and starch addition (right). Four independent test wells are shown for each strain B) Titration volume needed for each strain to reach the titration point. Values represent means ± standard deviation (n = 8).

The Winkler test validation for small sample volumes (1 ml) and its feasibility with high cell densities of algal cells were tested using the wild types CC-124 and CC-425 as positive controls and the Rubisco large subunit-deficient strain CC-2803 as a negative control. CC-2803, which produces less oxygen than it consumes, did not turn blue after the Winkler test, whereas the wild type needed to be titrated with sodium thiosulfate to reach the titration point (Figure 1). These results indicated that the Winkler test method is an appropriate way to select mutants with attenuated P/R ratios because it can discriminate between oxygen-evolving (P/R > 1) strains and oxygen-consuming mutants in which the P/R is < 1.

### Screening results and selection for hydrogen production strains under normal growth conditions

The two mutant libraries comprising about 9000 transformants were screened with the Winkler test. Out of those, three transformants (*apr*1, *apr*4 and *apr*6), which did not turn blue, and one transformant (*apr*7), which needed less Na_2_S_2_O_3 _than the wild type to reach the titration point, were selected for further physiological and biochemical studies (Figure 1). *Apr*1 was found in the CC-425/*Hind*III-pJD67 mutant library, whereas *apr*4, *apr*6 and *apr*7 derived from the CC-124 background strain transformed with the paromomycine resistance cassette.

**Figure 2:**
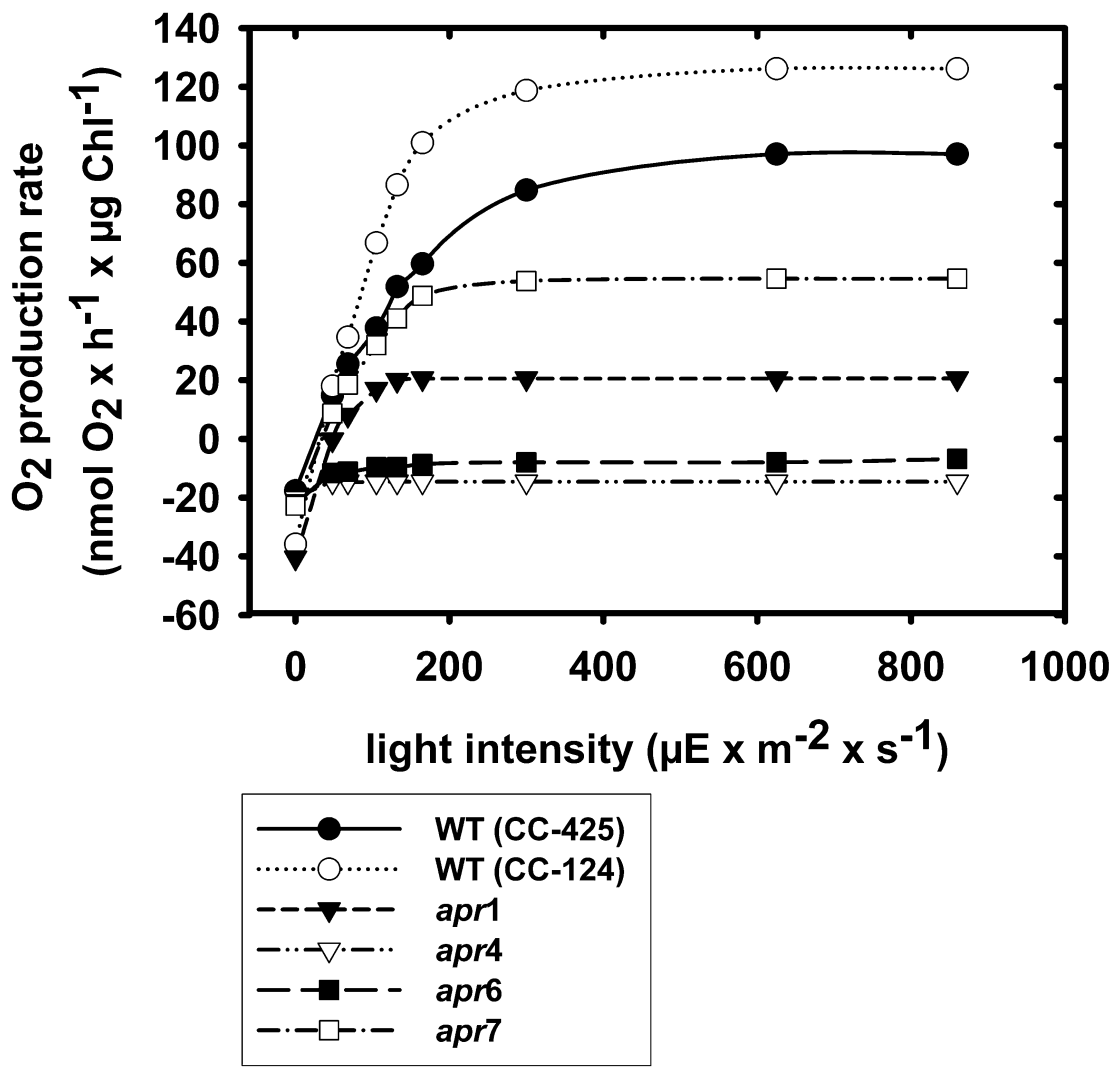
**Light saturation curves of the four *apr *mutant strains and the wild types CC-425 and CC-124**. Cell samples were examined in their linear growth phase in a Clark-type oxygen electrode. Oxygen production rates were determined at different light intensities, whereas respiration rates (displayed as values at a light intensity of 0 in the diagram) were recorded in the dark. Values represent means of three independent measurements.

**Figure 3:**
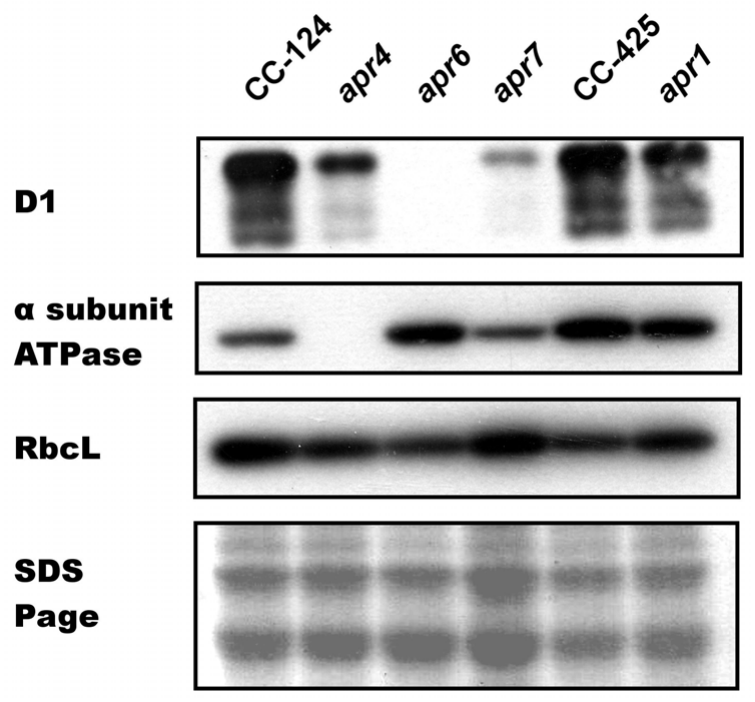
**Western blot analyses with antibodies against the core subunit D1 of PSII, the chloroplastic ATPase α subunit and the large subunit of Rubisco (RbcL)**. Protein samples of whole cell extract were loaded equally by adjusting them to the same chlorophyll concentration. A detail of a Coomassie stained SDS-Page is shown to verify equally loaded protein amounts. Protein extracts of the *C. reinhardtii *wild types CC-425 and CC-124 served as reference samples.

The light saturation curve of photosynthesis for each of the four mutant strains was recorded at the end of their exponential growth phase giving information about maximal oxygen production rates, respiration rates, and the compensation point (Figure 2). Additionally, the maximal quantum yield of PSII was calculated from fluorescence induction measurements for each mutant strain (Table 2). In order to elucidate physiological defects of the mutants, Western blot analyses with antibodies against the D1 core protein of PSII, the chloroplastic ATPase α-subunit, and the Rubisco large subunit RbcL were carried out (Figure 3).

**Table 2:** Physiological features of *C. reinhardtii apr *mutant strains and the respective wild types.

	***WT (CC-425)***	***WT (CC-124)***	***apr1***	***apr4***	***apr6***	***apr7***
P/R ratio at saturated light intensities	6.7 ± 1.7	4.2 ± 0.41	1.5 ± 0.1	0.6 ± 0.4	0.4 ± 0.1	3.5 ± 0.7
respiration rate (nmol O_2 _× h^-1 ^× μg Chl^-1^)	17.7 ± 3.2	35.6 ± 0.5	40.5 ± 4.9	14.7 ± 1.9	22.2 ± 2.7	22.7 ± 7.3
maximal O_2 _production rate (nmol O_2 _× h^-1 ^× μg Chl^-1^)	97.1 ± 13.8	114.8 ± 16.0	20.6 ± 5.2	-6.1 ± 5.9	-6.8 ± 0.7	54.6 ± 13.8
compensation point (μE × m^-2 ^× s^-1^)	27.2 ± 6.4	39.1 ± 6.8	53.1 ± 2.3	-	-	36.3 ± 5.1
light sensitive	no	no	no	yes	no	High light sensitive
Ability of photoautotrophic growth (TBP medium)	yes	yes	yes	no	no	yes
F_v_/F_m_	0.66 ± 0.01	0.66 ± 0.023	0.58 ± 0.02	0.54 ± 0.01	0.00 ± 0	0.30 ± 0.01

The compensation point reflects the light intensity at which the cultures produce as much oxygen as they consume. Exposing sealed cultures to lower light intensities than at the compensation point lead to anaerobiosis. Values represent means ± standard deviation (n = 3).

*Apr*1 showed a higher respiration rate (40.5 nmol O_2 _× h^-1 ^× μg Chl^-1^) and a lowered net oxygen production rate (20.6 nmol O_2 _× h^-1 ^× μg Chl^-1^) compared to the wild type CC-425. Calculating the ratio of the photosynthesis rate (the sum of the absolute value of the respiration rate and the net oxygen production rate) to the respiration rate resulted in a P/R ratio of 1.5 at saturating light intensities (850 μE × m^-2 ^× s^-1^). The compensation point of *apr*1 was reached at a light intensity of 53.1 μE × m^-2 ^× s^-1 ^which was higher than the respective light intensity for the wild type (27.2 μE × m^-2 ^× s^-1^). However, the maximal PSII quantum yields (0.58 for *apr*1 and 0.66 for CC-425) and protein levels examined by Western blot analyses did not differ significantly from the wild type (Table 2, Figure 3).

Mutant strain *apr*4 is light-sensitive, so that cells were always grown under dim light for the physiological and biochemical analyses. *Apr*4 displayed slightly lowered PSII maximal quantum yields than the wild type (0.54 for *apr*4 versus 0.66 for CC-124) but exhibited almost no photosynthetic oxygen evolution at all applied light intensities. Western blot analyses showed that strain *apr*4 misses the chloroplastic ATPase α subunit (Figure 3).

Permanently low oxygen production rates could also be measured in the *C. reinhardtii *transformant *apr*6. No functional PSII systems could be assumed from fluorescence measurements, and Western blot analyses showed that the PSII core protein D1 was missing (Table 2, Figure 3). Presumably, residual active PSII systems are still present in *apr*6 which are responsible for the low photolytic activity that could be detected from oxygen production measurements (Table 2).

*C. reinhardtii *strain *apr*7 showed the highest photosynthetic oxygen-evolving activity of all screened strains (54.6 nmol O_2 _× h^-1 ^× μg Chl^-1^), although the PSII quantum yield was lowered by half (0.3 for *apr*7 versus 0.66 for CC-124) and D1 levels were also reduced (Figure 3). Respiration rates were comparable to the control, and the compensation point was already reached at a light intensity of 36 μE × m^-2 ^× s^-1 ^which was comparable to the value measured in wild type cells.

Our aim was to analyze the hydrogen production routes during photosynthetic electron transport under steady state conditions. Thus, *C. reinhardtii *strain *apr*1 was selected for the studies on photosynthetic hydrogen metabolism. *Apr*1 established anaerobiosis under moderate light conditions (53.1 μE × m^-2 ^× s^-1^) without being severely affected in photosynthetic electron transport as indicated by the above-mentioned experiments and the fact that it could grow photoautotrophically (Table 2).

### *Apr*1 induces [FeFe]-hydrogenase synthesis under moderate light intensity and normal growth conditions, but does not evolve significant amounts of hydrogen

To examine the hydrogen metabolism of *C. reinhardtii *transformant *apr*1, cells were inoculated in sealed culture bottles in TAP medium and incubated under light intensities which were adjusted to be slightly lower than those determined to reach the compensation point. [FeFe]-hydrogenase enzyme activity was measured by *in vitro *activity assays, which detect active hydrogenase enzymes in whole-cell extracts supplied with reduced methylviologene as artificial electron donor for the [FeFe]-hydrogenases under oxygen-free conditions. The use of an artificial electron donor allows the detection of any active hydrogenase enzyme within the cells, independent from the physiological electron supply to the hydrogenase which usually limits net hydrogen production.

**Figure 4:**
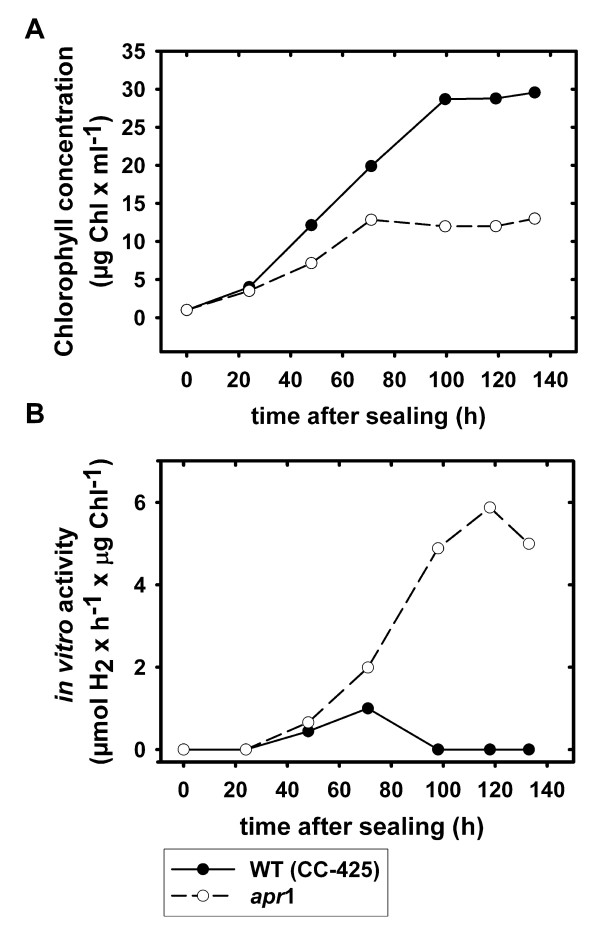
**Growth (A) and [FeFe]-hydrogenase *in vitro *activity (B) in *C. reinhardtii *wild type CC-425 and strain *apr*1 cultures in sulfur-replete TAP medium**. Cultures were sealed after inoculation (1 μg Chl × ml^-1^) and exposed to light intensities of 60 to 70 μE × m^-2 ^× s^-1^. Chlorophyll content (A) and *in vitro *[FeFe]-hydrogenase activity (B) were measured daily.

*Apr*1 *in vitro *[FeFe]-hydrogenase activity could be detected after the second day of growth under the above-mentioned conditions and rose to its maximal level after five days (Figure 4, panel B). Hence, sealed *apr*1 cultures established anaerobiosis without any external manipulation and synthesized [FeFe]-hydrogenases in parallel to cell proliferation.

However, despite high *in vitro *hydrogenase activities, no *in vivo *hydrogen production rates were detectable over the culture period. Only traces of hydrogen in the headspace of sealed *apr*1 could be measured by gas chromatography (data not shown). This observation indicated that the electron supply to the hydrogenase was limited.

### Calvin-Benson Cycle inactivation in *apr*1 by glycolaldehyde (GA)

GA disrupts Calvin-Benson cycle activity by inhibiting the phosphoribulokinase which catalyses the ATP-dependent phosphorylation of ribulose-5-phosphate to ribulose-1,5-bisphosphate. In former studies, GA was employed for the examination of carbon dioxide transport without concomitant carbon dioxide fixation, or for investigating the dependence of the PSII repair system on the activity of the Calvin-Benson cycle [25,26].

**Figure 5:**
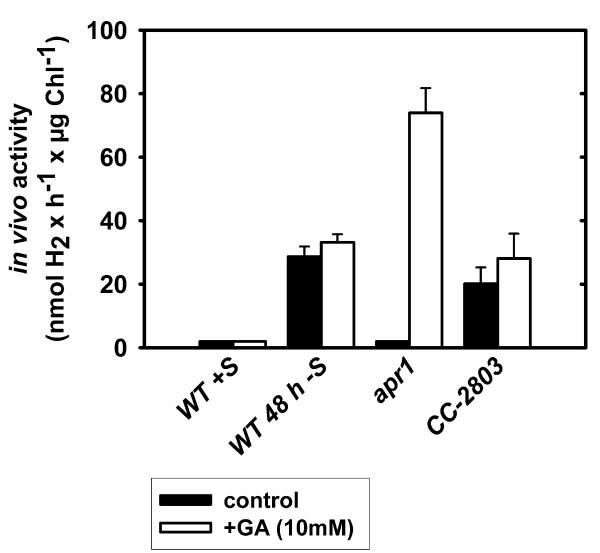
**Short-term effect of Calvin-Benson cycle inactivation on hydrogen production**. *In vivo *hydrogen production rates were determined upon Calvin-Benson cycle inactivation for the *C. reinhardtii *wild type in full medium (WT+S), for the wild type after 48 hours of sulfur depletion (WT 48 h -S), for the [FeFe]-hydrogenase-activated *apr*1 mutant and for the strain CC-2803 in full medium. Values represent means ± standard deviation (n = 3).

*C. reinhardtii *mutant strain *apr*1 becomes anaerobic under photoheterotrophic, sulfur-replete conditions and triggers [FeFe]-hydrogenase synthesis in the light as shown by *in vitro *activity assays (Figure 4). In order to analyze the influence of Calvin-Benson cycle activity on hydrogen metabolism under [FeFe]-hydrogenase-activated conditions, *in vivo *hydrogen production rates in cultures of *apr*1 were determined depending on GA treatment (Figure 5). As controls, the sulfur-deprived wild type and the Rubisco-deficient strain CC-2803 in full medium were treated with GA. Similar to *apr*1, CC-2803 can establish anaerobiosis and induces [FeFe]-hydrogenase synthesis under sulfur-replete conditions. However, this strain cannot fix carbon dioxide [14], which is in contrast to strain *apr*1. Upon GA addition, the *in vivo *hydrogen production rate of [FeFe]-hydrogenase-activated *apr*1 cell samples rose from 0 to 74 nmol × H_2 _× h^-1 ^× μg Chl^-1 ^and was thus double as high as the rate determined in sulfur-deprived wild type cells. Calvin-Benson cycle inhibition in sulfur-deprived wild type and CC-2803 cell samples did not result in a significant increase of hydrogen production capacity (Figure 5).

**Figure 6:**
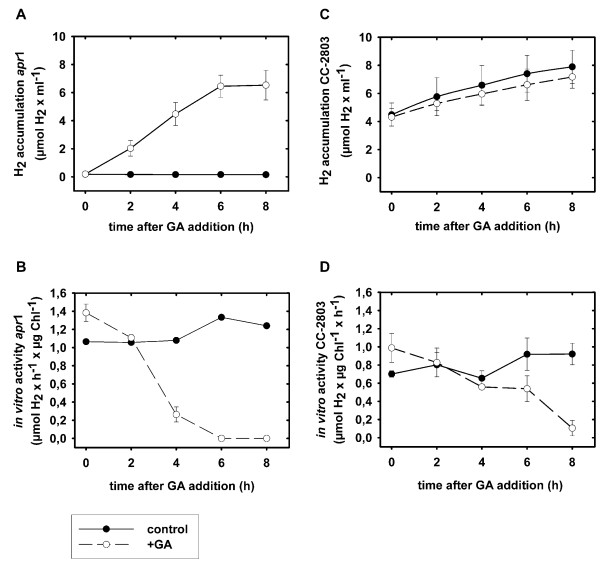
**Long term effects of glycolaldehyde (GA) addition on [FeFe]-hydrogenase activated *apr*1 and CC-2803 cultures**. *Apr*1 and CC-2803 cells were transferred into new TAP medium and adjusted to 20 μg × ml^-1 ^chlorophyll concentration. Cultures were sealed and exposed for twelve hours to 60 μE × m^-2 ^× s^-1 ^to induce [FeFe]-hydrogenase synthesis. Then, GA was added (time = 0 h in the diagrams) and hydrogen accumulation in the bottle headspace as well as *in vitro *hydrogenase activities were measured every two hours in GA-treated and in control cells. Values represent means ± standard deviation (n = 3)

Hydrogen accumulation of GA-treated, [FeFe]-hydrogenase-activated *apr*1 cultures lasted six hours under 60 to 70 μE × m^-2 ^× s^-1 ^incident actinic light exposure, and the hydrogen concentration rose from 0.19 to 6.5 μmol H_2 _× ml^-1 ^(Figure 6, panel A). Hydrogen production obviously stopped, because the amount of active hydrogenase enzymes, as determined by *in vitro *activity assays, declined steadily in the first four hours and ceased after six hours (Figure 6, panel B). Photosynthesis and respiration rates were strongly lowered by the presence of GA as well (data not shown).

Hydrogen concentrations in the headspaces of the CC-2803 control and GA-treated CC-2803 cultures did not differ significantly over the measuring period (Figure 5, panel C). Both cultures accumulated hydrogen almost to the same degree during eight hours. Similar to *apr*1, the *in vitro *hydrogenase activity of GA-treated CC-2803 cultures decreased and was undetectable after eight hours (Figure 5, panel D).

## Discussion

### A novel screening system for the identification of photosynthetic microorganisms that are altered in their photosynthesis-respiration balance was designed

One aim of this work was to establish an alternative approach for the induction of hydrogen metabolism in *C. reinhardtii*, so as to be able to achieve photosynthetic electron transport to the hydrogenase under steady state growth conditions. For this purpose, a selective screening system for mutants attenuated in their photosynthesis/respiration capacity ratio was established.

The screening system, which is based on the so-called Winkler test to measure dissolved oxygen concentrations, indeed captured a new class of mutant strains that can survive under photoautotrophic conditions (Table 2) but are attenuated in their P/R capacity ratio. This is particularly the case for the *C. reinhardtii *mutant strains *apr*1 and *apr*7.

However, in addition to reaching our actual goal, we found that the screening procedure allows the identification of a broad phenotypic spectrum of mutant strains. Besides the two mutants with attenuated P/R ratios, two acetate-requiring mutants were identified confirming the robustness of the screening procedure. Thus, the iodometric determination of dissolved oxygen concentrations is a cheap and fast method which can be employed universally for screenings of oxygenic microorganisms that are altered in their photosynthesis to respiration ratio and their photosynthetic activity, respectively. Another advantage of the method is that environmental conditions can be modified for the design of more selective screening protocols. For example, exposing the plates of a transformant library to high light would allow the finding of mutants with an imbalanced rate of PSII synthesis to PSII repair rate, whereas lower light intensities would result in screening of mutants with pronounced respiration rates.

From all screened mutants, *apr*1 was the most suitable to examine hydrogen production under normal growth conditions. Strains *apr*4 and *apr*6 are characterized by a P/R ratio lower than one and could thereby establish anaerobiosis under all light intensities (Figure 2). However, charge separation and linear electron transport were drastically altered in *apr*4 and *apr*6 (Table 2). In strain *apr*6, PSII was significantly affected, prohibiting the measurement of PSII-driven electron transport to the [FeFe]-hydrogenase. Strain *apr*4 misses a subunit of the plastidic ATP-synthase. Studies with ATPase deficient *C. reinhardtii *mutants which are not capable to assemble the thylakoid membrane localized coupling factor CF_0 _showed a progressive loss of PSII cores after several hours of medium light exposure [27]. Similar results could be obtained with the light-sensitive *apr*4. After *apr*4 had been exposed to medium light conditions, chlorophyll fluorescence measurements and immunodetection of D1 indicated a degradation of PSII cores as well (data not shown). Thus, in strain *apr*4, linear photosynthetic electron transport is strongly affected, both because of damaged PSII centres and an altered proton gradient.

Since hydrogen production is dependent on photosynthetically reduced ferredoxin, these non-photoautotrophic strains were not appropriate to analyze the hydrogen metabolism under normal growth conditions. Nevertheless, *apr*4 and *apr*6 are eligible candidates for the research field studying the regulation of PSII or ATPase subunit synthesis, assembly, or repair.

### Reductants are primarily directed to assimilative pathways in [FeFe]-hydrogenase-activated *apr*1 cultures

In the following studies we concentrated on *C. reinhardtii *mutant strain *apr*1, whose attenuated P/R ratio could be used to induce [FeFe]-hydrogenase synthesis by adjusting the light to moderate intensities as expected. However, *apr*1 did not produce significant amounts of hydrogen during the [FeFe]-hydrogenase-active phase (Figure 5).

Presumably, most of the photo-reduced ferredoxin is employed for assimilative pathways and NADPH generation, but not for hydrogen production under normal growth conditions, which is also indicated by the fact, that *apr*1 was still able to proliferate under our experimental conditions.

The ratio of reduced to oxidized ferredoxin displays a key junction between photosynthesis and anabolic pathways like sulfur/nitrogen/carbon assimilation or amino acid synthesis [28]. One major recipient of cellular reductants under normal growth condition is the ferredoxin-NADP^+ ^reductase which supplies the Calvin-Benson cycle with NADPH. Enzyme kinetic studies provided data about Michaelis-Menten constants (K_m_) of the *C. reinhardtii *[FeFe]-hydrogenase for spinach ferredoxin (35 μM) and of the *C. reinhardtii *FNR for ferredoxin coupled to cytochrome c reduction (2.5 μM) [29,30]. Obviously, even if active [FeFe]-hydrogenase is synthesized in growing *apr*1 cultures, the different Michaelis-Menten constants of ferredoxin for the [FeFe]-hydrogenase and for FNR result in a clear preference of passing photosynthetic electrons to assimilative pathways rather than to hydrogen evolution.

### Hydrogen metabolism as a protection system to dissipate excess photon energy in the absence of other electron sinks

We suggested that under normal growth conditions, even in the [FeFe]-hydrogenase-activated *apr*1 cells, photosynthetically provided electrons are utilized for assimilatory processes, carbon dioxide fixation above all.

It has been shown that hydrogen production in anaerobically adapted alga is highest when the carbon dioxide concentrations are lowest and *vice versa *[31]. A recent study showed that the Rubisco-deficient strain CC-2803, which also has a permanently reduced PSII activity, can produce hydrogen in complete medium [14]. It was discussed that in this strain the hydrogenase acts as an alternative electron sink in the absence of the Calvin-Benson cycle. Reversely we concluded that inhibition of carbon dioxide fixation would result in a net production of hydrogen in anaerobic *apr*1 cultures. Indeed, treatment of *C. reinhardtii *strain *apr*1 with GA yielded high *in vivo *hydrogen production rates that were even higher than in the sulfur-deprived *C. reinhardtii *wild type. On the other hand, the addition of GA to the Rubisco-deficient strain CC-2803 had no significant effect on hydrogen evolution, indicating that GA acts specifically on the activity of the Calvin-Benson cycle.

However, neither *in vitro *nor *in vivo *hydrogenase activity sustained longer than six hours under medium light exposure in the examined cells. No biochemical effect of GA on *in vitro *activity of purified [FeFe]-hydrogenase could be observed (data not shown). Obviously, GA has a deleterious effect on photosynthesis and an indirect physiological effect on [FeFe]-hydrogenase activity. One explanation is that the efficiency of the "hydrogenase valve" is not efficient enough so that the photosynthetic electron transport chain becomes over-reduced resulting in photoinactivation of PSII [25]. Alternatively, GA, which is also substrate of e.g. ketolases, has another deep impact on the cellular metabolism of *C. reinhardtii *[32]. Another explanation could be that the cellular concentration of active [FeFe]-hydrogenase enzymes decreases due to lowered protein biosynthesis rates [25].

Still, this study provides additional evidence for the model that hydrogen generation via [FeFe]-hydrogenase is a tool by which *C. reinhardtii *is able to dispose excess redox power and permits coupled electron transport in the thylakoid and mitochondrial membranes to generate ATP albeit only under microaerobic/anaerobic conditions [13]. The ATP energy helps the cell to survive under these stress conditions, when no CO_2 _fixation takes place.

In view of the natural habitats of *C. reinhardtii*, this strategy makes sense. Especially in freshwater ponds and puddles, macronutrients including carbon dioxide as well as light can become limited due to growth of other microorganisms. Under these low light conditions, photosynthetic activity would probably become low enough to allow the establishment of microaerobic/anaerobic conditions and the expression of the hydrogenase genes therewith.

## Conclusion

The Winkler test screening procedure is a valuable method for the identification of a new class of *C. reinhardtii *mutants which are characterized by an imbalanced P/R ratio, but which are still able to grow photoautotrophically. These mutants constitute a new platform to induce a hydrogen metabolism in the light under normal growth conditions without nutritional stress. Thus, such transformants allow the analysis of a steady state hydrogen metabolism. The results of strain *apr*1 clearly show that this class of strains is suited to get deeper information about the electron sources and sinks involved in hydrogen production. Furthermore, *C. reinhardtii *strains with a lowered P/R capacity ratio are promising to be used in biotechnological approaches as has been suggested by others [33]. However, the presented inhibitor studies showed that – besides balancing the P/R ratio in order to establish anaerobic/microaerobic conditions essential for [FeFe]-hydrogenase synthesis – other assimilative pathways have to be down regulated or inactivated for significant *in vivo *hydrogen production rates under normal growth condition.

## Methods

### Algal growth, sulfur deprivation

Algal cells were grown photoheterotrophically in TAP medium [34]. The arginine-auxotrophic CC-425 cultures were always supplied with filter-sterilized L-arginine to a final concentration of 10 μg × ml^-1^. Flasks were shaken (100 rpm) under moderate light conditions (50 to 70 μE × m^-2 ^× s^-1^) at 20°C. In case of the light-sensitive strains CC-2803 and *apr*4, cells were grown under very low light intensities (1–2 μE × m^-2 ^× s^-1^). Photoautotrophic growth was tested in modified TAP medium (TBP medium) in which the organic carbon source acetate was replaced by 25 mM sodium bicarbonate.

For sulfur deprivation, CC-124 cells were harvested in the linear growth phase (15 to 25 μg Chl^-1 ^× ml^-1^), washed once with TAP-S, and adjusted to a final chlorophyll concentration of 15 to 20 μg Chl × ml^-1^. TAP-S has the same medium composition as TAP, but the sulfate-containing salts are replaced by their chloride counterparts. In order to induce the hydrogen metabolism, sulfur-deprived cells were transferred into squared glass bottles, sealed with a gas-tight septum (red rubber Suba seals 37, Sigma-Aldrich, St. Louis, Mo, USA) and exposed to 60 to 70 μE × m^-2 ^× s^-1^.

### Mutant library construction

CC-425 was mutagenized with the *Hind*III-linearized, 12.3 kb long pJD67 plasmid [20]. Transformation was carried out with 9 × 10^7 ^cells and 2.5 μg linearized pJD67 plasmid. The second mutant library based on the CC-124 wild type which was transformed with a *aph*VIII gene construct coding for paromomycine resistance [21]. The *aph*VIII containing, 1.87 kb long fragment was obtained from a double endonuclease restriction (*Kpn*I, *Stu*I) of the pSL18 plasmid. For each glass bead transformation 800 ng of the 1.87 kb long resistance cassette and 6 × 10^7 ^autolysin-treated *C. reinhardtii *cells were employed[35].

### Winkler test screening protocol

Colonies from the mutant libraries were transferred to 48-well plates (Corning incorporated, costar^®^; Corning New York, total well volume of 1.6 ml) containing 200 μl TAP medium per well. The plates were exposed to low light for three or four days depending on the cell growth. Cells were supplied with 800 μl of fresh TAP medium, and a sterile solid glass bead (diameter 3 mm) was added to each well for optimal mixing. The plates were exposed to 40 to 80 μE × m^-2 ^× s^-1 ^for additional six hours. Next, the plates were incubated in the dark for 20 minutes in an anaerobic bag (I^2^R^® ^Glove Bag™, inflatable glove chamber model "X") which was continuously flushed with nitrogen gas. Alternatively, the plates were transferred into an anaerobic tent for further manipulations. After complete respiratory consumption of dissolved oxygen, the plates were exposed to 70 to 100 μE × m^-2 ^× s^-1 ^for 20 to 30 minutes. Photosynthetically evolved oxygen was fixed by adding successively 10 μl MnCl_2 _(0.34 M) and 10 μl KI/NaOH (0.24 M/1.2 M) in each well. After mixing, 50 μl H_3_PO_4 _(v/v 50%) was added to dissolve the precipitate. All subsequent steps were performed under aerobic conditions. In order to visualize iodine formation 10 μl 4% (w/v), starch solution was supplemented to the test solution giving a dark-blue color. Each well was titrated with Na_2_S_2_O_3 _(0.01 M) in 10 μl to 50 μl titration steps until the dark-blue color completely disappeared.

### Light saturation curves and chlorophyll measurements

Light saturation curves were measured with a Clark-type oxygen electrode (model respire 1 from Hansatech, Norfolk, UK). Actinic light was supplied by a projector lamp. The output signal was calibrated with oxygen-saturated water, and the baseline was adjusted by adding sodium dithionate which eliminated dissolved oxygen. Algal samples (2 ml) from their linear growth phase were transferred into the measurement chamber, and oxygen uptake rates were recorded in the dark for a few minutes until the rate appeared constant. The light intensity was increased successively every three minutes (45, 65, 105, 135, 165, 300, 625 and 860 μE × m^-2 ^× s^-1^). Assuming that oxygen-saturated water displays a concentration of 8.14 mg O_2 _× l^-1 ^at 20°C under atmospheric pressure, oxygen uptake and oxygen production rates were calculated on a per chlorophyll basis. For the determination of chlorophyll concentrations, cells were harvested, resuspended in 80% acetone and incubated for 20 minutes in the dark. Cell debris was removed by centrifugation and chlorophyll absorption of the supernatant was measured photospectrometrically at 652 nm (absorption coefficient: E = 27.8 μg Chl^-1 ^× cm^-1 ^× ml^-1^)

### Chlorophyll fluorescence measurements

Prior to chlorophyll fluorescence measurements, algal cells were incubated for at least five minutes in the dark. Samples were supplemented with DCMU (3-(3'-4'-dichlorophenyl)-1-1-dimethylurea) to a final concentration of 2.5 μM. The initial (F_0_), the variable (F_v_) and the maximum fluorescence yields (F_m_) were determined upon excitation with green light (75 μE × m^-2 ^× s^-1^) provided by a projector lamp covered with a red and a blue filter (CS 4–96 and CS 3–69 Corning Filters).

### Western blot analyses

Cell samples (containing 20 μg chlorophyll) were harvested and resuspended in 40 μl extraction buffer (0.1 M dithiothreitol and 1 M Na_2_CO_3_) and 40 μl solubilization buffer (250 mM Tris-HCl (pH 6.8), 7% (w/v) SDS, 20% (v/v) glycerol, 2 M urea, 10% (v/v) β-mercaptoethanol and 0.05% (w/v) bromphenol blue). Samples were incubated for approximately one hour at room temperature. After pelleting the cell debris, solubilized proteins in the supernatant were loaded on SDS polyacrylamide gels consisting of a 12% separation gel and a 4.5% stacking gel [36]. Loaded samples were adjusted to 0.5 μg for Western blot analyses and to 2 μg chlorophyll for Coomassie staining. After gel electrophoresis, proteins were transferred onto polyvinylidine difluoride membranes (PVDF) by electroblotting (50 mA for 20 hours; transfer buffer: 25 mM Tris, 190 mM glycine and 20% (v/v) methanol). Membranes were washed in 1× TBST buffer (10 mM Tris pH 8, 150 mM NaCl, 0.05% (v/v) Tween 20), and subsequent blocking of the membrane was performed in TBST supplemented with 5% (w/v) skim milk for one hour. After first antibody treatment for one hour in TBST supplemented with 5% (w/v) skim milk and three times washing with TBST, membranes were blocked again for one hour. Upon secondary antibody treatment for one hour (horseradish peroxidase conjugated secondary antibodies from Bio-Rad, Helcules/California) and four washing steps in TBST, hybridization signals were detected with the Supersignal ECL (Pierce, Rockford, IL, USA) detection kit according to the manufacturer's instructions.

For Coomassie staining SDS gels were incubated for one hour in a staining solution containing 0.25% (w/v) Coomassie R-250, 45% (v/v) ethanol and 45% (v/v) acetic acid. Destaining was carried out overnight with a destaining solution composed of 4.5% (v/v) ethanol and 4.5% (v/v) acetic acid.

### [FeFe]-hydrogenase *in vitro *activity assay

The [FeFe]-hydrogenase activity of cell samples was measured in presence of the artificial electron donor methylviologene under oxygen-free condition. The final reaction solution (2 ml) was composed of 100 mM sodium dithionate, 1% (v/v) triton X-100, 10 mM methylviologene and 60 mM potassium phosphate buffer (pH 6.8). Reaction vessels (8 ml volume) containing triton X-100, potassium phosphate buffer and methylviologene were closed with Suba seals and flushed with argon to eliminate dissolved oxygen and oxygen in the headspace. Oxygen-free sodium dithionite was added with a syringe to the [FeFe]-hydrogenase *in vitro *assay for methylviologene reduction. 0.5 ml cell samples were transferred with a syringe into the vessels containing the detergent triton X-100 and vortexed for ten seconds to disrupt the cells. The solutions were argon-flushed again for one minute to eliminate residual dissolved oxygen or hydrogen and placed into a water bath at 37°C under continuous shaking (150 rpm) for 20 minutes. The gas composition of 200 μl of the vessel headspace was analyzed with a gas chromatograph (GC-2010, Shimadzu, Kyoto, Japan; equipped with a PLOT fused silica coating molsieve column [5Å, 10 m by 0.32 mm] from Varian, Palo Alto, CA). The *in vitro *[FeFe]-hydrogenase activity was calculated on a per chlorophyll basis.

### [FeFe]-hydrogenase *in vivo *activity assay

In order to prevent oxygen inhibition of active [FeFe]-hydrogenases, 1 ml cell samples were transferred with a syringe into gas-tight vessels (total volume 8 ml) in which the air had been replaced by argon. In case of glycolaldehyde inhibition, cells were directly injected into the oxygen-free vessels containing the inhibitor (10 mM final concentration). Samples were flushed with argon for one minute and placed into a 20°C water bath for one hour under continuous shaking (150 rpm) and exposure to moderate light intensities (60 to 80 μE × m^-2 ^× s^-1^). The amount of evolved hydrogen in the vessel headspace was determined by gas chromatography in the same way as mentioned above for the [FeFe]-hydrogenase *in vitro *activity assay. Hydrogen evolution rates were related to the total chlorophyll content in the vessel.

### Glycolaldehyde inhibition of [FeFe]-hydrogenase activated *apr*1 and CC-2803 cultures

*C. reinhardtii *strains *apr*1 and CC-2803 were harvested in their linear growth phase (10 to 20 μg × ml^-1 ^chlorophyll), resuspended in fresh TAP+S medium to 20 μg × ml^-1 ^chlorophyll, and transferred into squared 120 ml glass bottles which were tightly plugged with suba seals. The cells were placed into the light (60 μE × m^-2 ^× s^-1^) for 12 to 15 hours under continuous stirring. GA was dissolved in water and transferred into plugged vessels which were argon-flushed for ten minutes to eliminate dissolved oxygen. To inactivate the Calvin-Benson cycle of [FeFe]-hydrogenase-activated cultures, anaerobic GA solution was transferred with a syringe to a total concentration of 10 mM.

## Authors' contributions

TR developed the experimental design, conducted the experiments and drafted the manuscript. AH participated in designing the inhibitor studies and in manuscript drafting. AM conceived of the study and participated in its design and coordination. TH conceived of the study and participated in its design and coordination. All authors read and approved the final manuscript.
